# Time trends in the impact factor of Public Health journals

**DOI:** 10.1186/1471-2458-5-24

**Published:** 2005-03-18

**Authors:** Gonzalo López-Abente, Concha Muñoz-Tinoco

**Affiliations:** 1 National Center for Epidemiology, Carlos III Institute of Health, Sinesio Delgado 6, 28029 Madrid, Spain; 2 Ramón y Cajal Hospital Library, Ctra. de Colmenar km 9.100, 28034 Madrid, Spain

## Abstract

**Background:**

Journal impact factor (IF) is linked to the probability of a paper being cited and is progressively becoming incorporated into researchers' *curricula vitae*. Furthermore, the decision as to which journal a given study should be submitted, may well be based on the trend in the journal's overall quality. This study sought to assess time trends in journal IF in the field of public, environmental and occupational health.

**Methods:**

We used the IFs of 80 public health journals that were registered by the Science Citation Index from 1992 through 2003 and had been listed for a minimum period of the previous 3 years. Impact factor time trends were assessed using a linear regression model, in which the dependent variable was IF and the independent variable, the year. The slope of the model and its statistical significance were taken as the indicator of annual change.

**Results:**

The IF range for the journals covered went from 0.18 to 5.2 in 2003. Although there was no statistical association between annual change and mean IF, most of the fastest growing journals registered mean IFs in excess of 1.5, and some represented emerging areas of public health research. Graphs displaying IF trends are shown.

**Conclusion:**

In view of the delay between the publication of IFs and that of any given paper, knowing the trend in IF is essential in order to make a correct choice of journal.

## Background

Scientific journal impact factor (IF) is directly linked to the probability of a paper being cited. It is currently accepted that a higher IF indicates a better journal quality, existing a correlation among IF and quality indicators at least in some health disciplines [1]. As a result these indices are progressively becoming incorporated into researchers' *curricula vitae*. In Spain, publication in top-IF journals has occupational implications in terms of academic careers and obtaining research grants [2]. The most widespread and important bibliometric indicators are those referring to the repercussion of scientific activity, and among these, IF has a leading role [3].

This pressure means that when it comes to having to select a journal in which to publish their studies, researchers turn to journals with IF, and assess the possibility of publishing in those that have the highest IF possible. Journals having the top IF within each medical specialty tend to be those with the greatest international prestige and highest profile, i.e., the most widely read by researchers and most in demand to publish their studies. However, the publication of extraordinary relevant scientific findings are concentrated in a small list of core journals, most of them not directly related with the specialty of the author [1,4]

Positive evaluation of IF which failed to take account of its trend over time would tend to favor publications of recent, but not necessarily lasting, interest. In contrast, publications of steadily growing interest and stable impact would be undervalued, though there may be differences of opinion about this [5].

Hence, in addition to journal quality, the decision as to which journal a manuscript should be submitted may be based on the trend in its IF over time. The aim of this study was to analyze IF time trends of journals in the "Public, Environmental and Occupational Health" category, thereby furnishing a new criterion on which to base the choice of journal for publication.

## Methods

For study purposes, we selected 80 journals that: were included in the "Public, Environmental and Occupational Health" category of the hard copy version of the Journal Citation Reports (JCR) from 1992 through 2003 [6]; were listed for a minimum period of 3 consecutive years; and had IFs in the JCR for 2003. We consulted the 2003 JCR-IFs via the ISI Web of Knowledge [7].

The impact factor is one of the quantitative tools provided by JCR for ranking, evaluating, categorizing, and comparing journals. The annual impact factor of a journal is calculated by dividing the number of current year citations to the source items published in that journal during the previous two years [6].

Impact factor time trends were assessed using a linear regression model, in which the dependent variable was IF and the independent variable, the year. The slope of the model (index of annual change-IAC) and its statistical significance were taken as the indicator of year-to-year variation.

## Results

Shown in Table 1 is a list of all journals included, ranked by their impact factor in 2003. This table also shows the index of annual change (slope of the model) and its statistical significance. Table 2 shows the same list, but ranked this time according to the IAC. This method of ranking can prove useful, since, by comparing the IF trends between two journals with similar IFs, the better choice would be the journal with the better index of annual change.

**Table 1:** Journals ranked by Impact Factor (IF) in 2003

Title	IAC	IF(2003)	mean	p-value
ANNU. REV. PUBL. HEALTH	0.225	5.179	3.158	0.010
CANCER. EPIDEMI. BIOMAR	0.214	4.720	3.475	0.001
AM. J. EPIDEMIOL	0.099	4.486	3.788	0.000
EPIDEMIOLOGY	0.250	4.220	3.093	0.000
AM. J. PUBLIC. HEALTH	0.076	3.363	3.057	0.010
EPIDEMIOL. REV	-0.175	3.306	3.203	0.076
INT. J. EPIDEMIOL	0.125	3.289	1.820	0.001
AM. J. PREV. MED	0.232	3.256	1.440	0.000
TOBACC. CONTROL	0.509	3.164	2.052	0.181
MED. CARE	0.105	3.152	2.379	0.004
ENVIRON. HEALTH. PERSP	0.220	3.038	2.192	0.000
DRUG. SAFETY	0.238	2.971	2.059	0.000
CANCER. CAUSE. CONTROL	0.087	2.726	2.623	0.061
B. WORLD. HEALTH. ORGAN	0.115	2.442	1.838	0.001
ANN. EPIDEMIOL	0.141	2.345	1.995	0.001
J. EPIDEMIOL. COMMUN. H	0.075	2.332	1.679	0.003
PSYCHIATR. SERV	0.138	2.274	1.658	0.004
GENET. EPIDEMIOL	0.018	2.265	1.681	0.544
J. CLIN. EPIDEMIOL	0.065	2.227	1.872	0.001
TROP. MED. INT. HEALTH	0.181	2.156	1.477	0.003
TR. ROY. SOC. TROP. MED. H	0.064	2.114	1.553	0.001
AM. J. TROP. MED. HYG	0.019	2.105	1.950	0.058
QUAL. LIFE. RES	-0.171	2.000	2.089	0.149
INFECT. CONT. HOSP. EP	0.081	1.951	2.074	0.035
PREV. MED	0.013	1.889	1.540	0.568
J. MED. SCREEN	-0.033	1.867	1.815	0.696
OCCUP. ENVIRON. MED	0.100	1.847	1.755	0.013
SCAN. J. WORK. ENV. HEA	0.075	1.816	1.433	0.001
NEUROEPIDEMIOLOGY	0.095	1.762	1.411	0.001
J. ADOLESCENT. HEALTH	0.082	1.674	1.361	0.000
PAEDIATR. PERINAT. EP	0.132	1.673	1.176	0.005
J. WOMEN. HEALTH. GEN. B	0.388	1.561	0.928	0.007
AM. J. IND. MED	0.049	1.542	1.256	0.001
EPIDEMIOL. INFECT	0.023	1.509	1.594	0.199
J. OCCUP. ENVIRON. MED	0.081	1.472	1.349	0.121
QUAL. HEALTH. CARE	0.056	1.466	1.232	0.221
J. AEROSOL. MED	0.056	1.459	0.818	0.006
ENVIRON. RES	0.058	1.452	1.390	0.068
INT. ARCH. OCC. ENV. HEA	0.026	1.388	1.086	0.072
ANN. OCCUP. HYG	0.070	1.357	1.041	0.002
J. URBAN. HEALTH	0.316	1.286	0.723	0.002
EUR. J. PUBLIC. HEALTH	0.002	1.281	1.044	0.983
J. EXPO. ANAL. ENV. EPID	0.124	1.263	1.033	0.001
PALLIATIVE. MED	-0.103	1.185	1.627	0.060
PUBLIC. HEALTH. REP	0.025	1.139	1.012	0.192
STAT. MED	0.030	1.134	1.238	0.094
PATIENT. EDUC. COUNS	0.103	1.130	0.774	0.001
COMUNITY. DENT. ORAL	0.069	1.100	0.976	0.002
INT. J. HYG. ENVIR. HEAL	0.302	1.085	0.822	0.142
J. OCCUP. HEALTH	-0.040	1.047	1.049	0.445
SCAN. J. PUBLIC. HEALT	0.207	1.018	0.714	0.044
ANN. TROP. MED. PARASIT	0.052	1.010	0.837	0.000
J. PUBLIC. HEALTH. DENT	-0.010	1.000	0.787	0.568
J. PUBLIC. HEALTH. MED	0.032	0.973	0.805	0.014
EUR. J. EPIDEMIOL	0.022	0.972	0.676	0.080
AVIAT. SPACE. ENVIR. MD	0.075	0.946	0.681	0.007
FLUORIDE	0.034	0.907	0.560	0.018
ANN. HUM. BIOL	0.026	0.885	0.787	0.001
ARCH. ENVIRON. HEALTH	-0.054	0.878	1.391	0.028
J. SCHOOL. HEALTH	0.039	0.868	0.688	0.185
ANN. AGR. ENV. MED	0.216	0.827	0.590	0.065
HEALTH. PHYS	0.012	0.777	0.865	0.385
J. ENVIRON. SCI. HEAL. B	-0.034	0.758	0.718	0.131
INT. J. TECHNOL. ASSESS	0.013	0.754	0.922	0.686
SOZ. PREVENTIV. MED	0.170	0.750	0.525	0.013
PUBLIC. HEALTH	0.025	0.697	0.522	0.010
OCCUP. MED. OXFORD	0.041	0.693	0.464	0.010
BIOMED. ENVIRON. SCI	-0.036	0.609	0.557	0.596
AIHAJ	0.180	0.601	0.449	0.166
INT. J. ENVIRON. HEAL. R	0.094	0.588	0.419	0.088
ENVIRON. GEOCHEM. HLTH	0.027	0.565	0.369	0.082
INDOOR. BUILT. ENVIRON	0.085	0.525	0.496	0.542
TOXICOL. IND. HEALTH	0.106	0.508	1.051	0.206
REV. EPIDEMIOL. SANTE	0.024	0.485	0.401	0.003
IND. HEALTH	0.015	0.474	0.497	0.262
TROP. DOCT	0.022	0.347	0.326	0.089
J. ENVIRON. HEALTH	0.021	0.341	0.228	0.007
J. PUBLIC. HEALTH. POL	-0.023	0.314	0.615	0.675
WILD. ENVIRON. MED	-0.019	0.280	0.339	0.822
B. SOC. PATHOL. EXOT	-0.092	0.183	0.262	0.154

IAC = index of annual change

**Table 2:** Journals ranked in descending order, by index of annual change (IAC).

Title	IAC	IF(2003)	mean	p-value
TOBACC. CONTROL	0.509	3.164	2.052	0.181
J. WOMEN. HEALTH. GEN. B	0.388	1.561	0.928	0.007
J. URBAN. HEALTH	0.316	1.286	0.723	0.002
INT. J. HYG. ENVIR. HEAL	0.302	1.085	0.822	0.142
EPIDEMIOLOGY	0.250	4.220	3.093	0.000
DRUG. SAFETY	0.238	2.971	2.059	0.000
AM. J. PREV. MED	0.232	3.256	1.440	0.000
ANNU. REV. PUBL. HEALTH	0.225	5.179	3.158	0.010
ENVIRON. HEALTH. PERSP	0.220	3.038	2.192	0.000
ANN. AGR. ENV. MED	0.216	0.827	0.590	0.065
CANCER. EPIDEMI. BIOMAR	0.214	4.720	3.475	0.001
SCAN. J. PUBLIC. HEALT	0.207	1.018	0.714	0.044
TROP. MED. INT. HEALTH	0.181	2.156	1.477	0.003
AIHAJ	0.180	0.601	0.449	0.166
SOZ. PREVENTIV. MED	0.170	0.750	0.525	0.013
ANN. EPIDEMIOL	0.141	2.345	1.995	0.001
PSYCHIATR. SERV	0.138	2.274	1.658	0.004
PAEDIATR. PERINAT. EP	0.132	1.673	1.176	0.005
INT. J. EPIDEMIOL	0.125	3.289	1.820	0.001
J. EXPO. ANAL. ENV. EPID	0.124	1.263	1.033	0.001
B. WORLD. HEALTH. ORGAN	0.115	2.442	1.838	0.001
TOXICOL. IND. HEALTH	0.106	0.508	1.051	0.206
MED. CARE	0.105	3.152	2.379	0.004
PATIENT. EDUC. COUNS	0.103	1.130	0.774	0.001
OCCUP. ENVIRON. MED	0.100	1.847	1.755	0.013
AM. J. EPIDEMIOL	0.099	4.486	3.788	0.000
NEUROEPIDEMIOLOGY	0.095	1.762	1.411	0.001
INT. J. ENVIRON. HEAL. R	0.094	0.588	0.419	0.088
CANCER. CAUSE. CONTROL	0.087	2.726	2.623	0.061
INDOOR. BUILT. ENVIRON	0.085	0.525	0.496	0.542
J. ADOLESCENT. HEALTH	0.082	1.674	1.361	0.000
INFECT. CONT. HOSP. EP	0.081	1.951	2.074	0.035
J. OCCUP. ENVIRON. MED	0.081	1.472	1.349	0.121
AM. J. PUBLIC. HEALTH	0.076	3.363	3.057	0.010
AVIAT. SPACE. ENVIR. MD	0.075	0.946	0.681	0.007
J. EPIDEMIOL. COMMUN. H	0.075	2.332	1.679	0.003
SCAN. J. WORK. ENV. HEA	0.075	1.816	1.433	0.001
ANN. OCCUP. HYG	0.070	1.357	1.041	0.002
COMUNITY. DENT. ORAL	0.069	1.100	0.976	0.002
J. CLIN. EPIDEMIOL	0.065	2.227	1.872	0.001
TR. ROY. SOC. TROP. MED. H	0.064	2.114	1.553	0.001
ENVIRON. RES	0.058	1.452	1.390	0.068
J. AEROSOL. MED	0.056	1.459	0.818	0.006
QUAL. HEALTH. CARE	0.056	1.466	1.232	0.221
ANN. TROP. MED. PARASIT	0.052	1.010	0.837	0.000
AM. J. IND. MED	0.049	1.542	1.256	0.001
OCCUP. MED. OXFORD	0.041	0.693	0.464	0.010
J. SCHOOL. HEALTH	0.039	0.868	0.688	0.185
FLUORIDE	0.034	0.907	0.560	0.018
J. PUBLIC. HEALTH. MED	0.032	0.973	0.805	0.014
STAT. MED	0.030	1.134	1.238	0.094
ENVIRON. GEOCHEM. HLTH	0.027	0.565	0.369	0.082
ANN. HUM. BIOL	0.026	0.885	0.787	0.001
INT. ARCH. OCC. ENV. HEA	0.026	1.388	1.086	0.072
PUBLIC. HEALTH	0.025	0.697	0.522	0.010
PUBLIC. HEALTH. REP	0.025	1.139	1.012	0.192
REV. EPIDEMIOL. SANTE	0.024	0.485	0.401	0.003
EPIDEMIOL. INFECT	0.023	1.509	1.594	0.199
EUR. J. EPIDEMIOL	0.022	0.972	0.676	0.080
TROP. DOCT	0.022	0.347	0.326	0.089
J. ENVIRON. HEALTH	0.021	0.341	0.228	0.007
AM. J. TROP. MED. HYG	0.019	2.105	1.950	0.058
GENET. EPIDEMIOL	0.018	2.265	1.681	0.544
IND. HEALTH	0.015	0.474	0.497	0.262
INT. J. TECHNOL. ASSESS	0.013	0.754	0.922	0.686
PREV. MED	0.013	1.889	1.540	0.568
HEALTH. PHYS	0.012	0.777	0.865	0.385
EUR. J. PUBLIC. HEALTH	0.002	1.281	1.044	0.983
J. PUBLIC. HEALTH. DENT	-0.010	1.000	0.787	0.568
WILD. ENVIRON. MED	-0.019	0.280	0.339	0.822
J. PUBLIC. HEALTH. POL	-0.023	0.314	0.615	0.675
J. MED. SCREEN	-0.033	1.867	1.815	0.696
J. ENVIRON. SCI. HEAL. B	-0.034	0.758	0.718	0.131
BIOMED. ENVIRON. SCI	-0.036	0.609	0.557	0.596
J. OCCUP. HEALTH	-0.040	1.047	1.049	0.445
ARCH. ENVIRON. HEALTH	-0.054	0.878	1.391	0.028
B. SOC. PATHOL. EXOT	-0.092	0.183	0.262	0.154
PALLIATIVE. MED	-0.103	1.185	1.627	0.060
QUAL. LIFE. RES	-0.171	2.000	2.089	0.149
EPIDEMIOL. REV	-0.175	3.306	3.203	0.076

The IF range for the journals covered went from 0.18 to 5.2 in 2003. Although there was no statistical association between annual change and mean IF, most of the fastest growing journals registered mean IFs in excess of 1.5 (Journal of Womens Health and Gender Based Medicine IAC = 0.388 p = 0.007, Journal of Urban Health IAC = 0.316 p = 0.002, Epidemiology IAC = 0.250 p < 0.001, Drug Safety IAC = 0.238 p < 0.001), and some represented emerging areas of public health research (Table 2).

**Figure 1:**
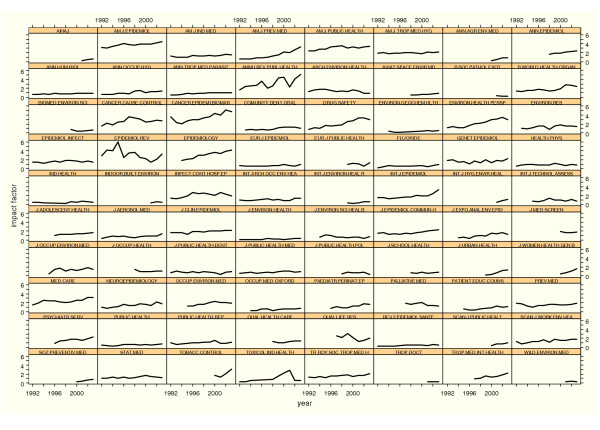
Time trends in the impact factor of Public Health journals by alphabetical order.

Figure 1 depicts IF trends on a multiple graph, thereby allowing a quick idea to be formed of the evolution of the indicator in all the journals included.

If journals that publish review papers are excluded, then the "American Journal of Epidemiology" ranked first in 1991, a position occupied in 1992 by a recently created publication with a fairly specific content matter, the "Cancer Epidemiology Biomarkers & Prevention", in tandem with a journal of similar orientation and seniority, viz., "Cancer Causes and Control". The rise of both these journals may be indicative of current priorities in epidemiologic research [8,9]. This upward trend pattern among journals addressing cancer epidemiology remained in evidence throughout the study period.

In 2003 the epidemiology journals with the highest IF ranged from 1.5 through 4.5, though it should be stressed that special caution is called for when dealing with the individual journal lists for the respective medical specialties.

## Discussion

The use of IF as an indicator of a journal's profile or prestige has become widespread among researchers, editors, libraries, and even among the agencies that fund research. Nevertheless, this indicator has a number of limitations that have been extensively debated in the literature [5,10]. Thus, for instance, journals that publish review papers receive a high number of citations and their impact factors are particularly high. It should be pointed out here that the ISI seeks to offer an overview of international science, with the result that journals covering topics or disciplines of more local interest are scarcely covered. Within each category, therefore, it is frequent for journals that are more basic -and thus of universal interest- to be associated with a higher impact factor than those that are more applied -and so of more local interest- given that the latter's circulation is more restricted. The publication of extraordinary relevant scientific findings are concentrated in a small list of core journals, most of them not directly related with the specialty of the author [1,4]. The mere quality of the documents published by a journal, albeit essential, will not suffice for it to be cited. The number of citations can be enhanced using management techniques, such as expanding a journal's international circulation, raising its profile in databases and on web pages, and increasing the number of papers. It becomes necessary for journals to be known among the international community and attain sufficient prestige to be subsequently cited. The policy pursued in Spain in recent years aimed at fostering high-quality, competitive science has induced Spanish scientists to bypass Spanish journals and send their publications instead to journals enjoying a wide international circulation, something that is often associated with journals having the greatest IF.

Our results suggest that it would be of interest to add the index of annual change to the criteria used for selecting a journal for publication. Studies conducted in another JCR area have shown the importance of analyzing IF trends by category [11]. IF trend might, however, be determined by certain factors that should be discussed.

The journals analyzed may be assigned to more than two categories in the SCI-JCR. The Public Health category changed names in 1996, and from 1997 onwards was called "Public, Environmental and Occupational Health", thus explaining why the number of titles jumped from 61 to 73 from one year to the next (Table 3).

**Table 3:** Trend in the number of journals classified in the Public, Environmental and Occupational Health category.

Year	1992	1993	1994	1995	1996	1997	1998	1999	2000	2001	2002	2003
No. of journals	58	60	58	60	61	73	80	84	87	88	90	89

Similarly, until the year 2000, the "Epidemiology" category came within the umbrella of "Public Health", thereafter disappearing and falling within the category of study without any specific entry. The categories covered by the JCR have changed in name and number over the years and, logically, this is equally true of the journals that comprise them.

In addition to the comments regarding the list of journals included in the category of study, it would be a wise decision on the ISI's part if allocation of journals to categories were made in agreement with the researchers [9]. However, it is not the intention of this paper to dwell on the use and abuse of the IF or the arbitrariness of the study-category journal list, an aspect previously analyzed in connection with the public health sphere [10].

This study solely included journals listed for 3 years in the JCR. Recently launched journals with a policy geared to novel publication, such as the BMC group, were not taken into consideration. The "BMC Public Health" journal has been listed for two years, with IFs of 0.29 in 2002 and 0.93 in 2003, and plots a growth pattern similar to that of journals addressing emerging issues.

In general terms and in view of Figure 1, the linear model seems to be adequate, inasmuch as there are very few journals with trends that display evident turning points. Many of the journals maintain their trend over the years. Most of them (60%) add 0.03 points or more to their IFs every year and there are very few that register a decline with time; indeed this is statistically significant in only one instance. A number of categories can be drawn up based on the trend pattern, namely: long-standing journals with the top IF, which maintain their trend; new journals focused on emerging issues, which seem to enjoy good acceptance; journals that maintain a very stable intermediate ranking; and a small group that has witnessed a decrease in their respective IFs.

The reasons why a journal changes its IF trend has been commented before and some of them does not have any relationship with a better quality of its papers. Probably a better or worst management of the journal also is related with the changes in the citations trend and could deserve some study.

In the publication of a scientific paper, a long time elapses between deciding upon a journal and the date of publication: on average, more than one year can go by. In view of the stability of the indicators in the area of study targeted, relying upon IF time trends in order to choose a particular journal might perhaps not be very relevant. Yet it may be a critical aspect in other areas of science where there are increases of around one point per year. For researchers/authors who know only too well how costly it can prove to see their paper published and are, moreover, aware that it is going to be evaluated on the basis of concepts as abstract as the impact and number of their publications, this decision is important.

## Conclusion

Leaving aside the speculative components of the choice, and given the delay that accumulates between the publication of IFs and that of any given paper, knowing the trend in the IF is yet another factor that will help authors make the correct choice of journal.

## Competing interests

The author(s) declare that they have no competing interests.

## Authors' contributions

CMT was responsible for the development of intellectual content and the study design, collected the data, data coding and entry, interpretation of the results, manuscript drafting and the critical revisions of manuscript. GLA was responsible for the development of intellectual content, statistical analyses, interpretation of the results and manuscript drafting. All authors read and approved the final manuscript.

## Pre-publication history

The pre-publication history for this paper can be accessed here:


